# When bariatric surgery reduces food addiction: a prospective study

**DOI:** 10.3389/fnut.2025.1535911

**Published:** 2025-11-27

**Authors:** Xu-Yan Ban, Chen Wang, Ting Xu, Lin Liu, Chuan-Yu Yin, Hui-Lin Zhang, Hong-Wei Zhang, Xiao-Dong Han, Hui Zheng, Jian-Zhong Di

**Affiliations:** 1 Department of Metabolic & Bariatric Surgery, Shanghai Sixth People's Hospital Affiliated to Shanghai Jiao Tong University School of Medicine, Shanghai, China; 2 Department of Gastrointestinal Surgery, Shanghai Fourth People’s Hospital, School of Medicine, Tongji University, Shanghai, China; 3 College of Fisheries and Life Science, Shanghai Ocean University, Shanghai, China; 4 Neuroimaging Research Branch, National Institute on Drug Abuse, National Institutes of Health, Baltimore, MD, United States

**Keywords:** food addiction, bariatric surgery, YFAS 2.0, mixed linear model, cluster analysis

## Abstract

**Background:**

Studies have shown that patients with obesity appear to be more susceptible to food addiction than the general population. Bariatric surgery stands as the most potent remedy for combating obesity, and it is believed to alleviate the manifestations of food addiction. However, the timing of bariatric surgery to improve food addiction has seldom been the focus of attention.

**Methods:**

In this research, 78 individuals who underwent bariatric surgery were tracked over a period of 2 years. The Yale Food Addiction Scale 2.0 (YFAS 2.0) was employed to assess changes in food addiction tendencies post-surgery. Mixed linear modeling and cluster analysis were applied to investigate the timing of influence of bariatric surgery on the evolution of sub domains of food addiction.

**Results:**

We found that: (1) Bariatric surgery significantly reduces food addiction scores; (2) Bariatric surgery rapidly reduces food addiction scores within first month of surgery and extends to 2 years after surgery; (3) Symptoms in the YFAS 2.0 could be divided into two domains (rapid decline / slow decline) based on their progression following surgery. Rapid decline domain experience rapid improvement shortly (usually 1st month) after the bariatric surgery and maintains a consistently low symptom level, while the slow decline domain improves slowly (usually 4th month) in the post-operative phase.

**Discussion:**

Bariatric surgery induced rapid and sustained remission of food addiction, significantly reducing total food addiction scores within 1 month postoperatively. The effects maintained through 24 months, potentially through neurophysiological and gut microbiota alterations. Despite rapid remission of most food addiction symptoms, social/interpersonal problems, hazardous use, and large amount/longer showed delayed improvement, suggesting distinct behavioral persistence mechanisms.

**Conclusion:**

Food addiction scores can rapidly decline postoperatively and remain consistently lower. However, bariatric surgery does not fully improve all addiction symptoms at the 1st month. This suggests the importance of establishing multidisciplinary clinics in bariatric metabolic surgery.

## Introduction

Obesity is a pervasive and costly public health crisis (1), with higher prevalence of complications such as psychological disorders, such as anxiety (2), depression (3) and eating behavior disorders (4, 5). Bariatric surgery is the most effective form of treatment for patients with severe obesity and associated complications (6). However, not all those who have bariatric surgery can solve these complications, especially complex eating behavior disorders. Such as food addition, anorexia, bulimia, before and after surgery, can lead to weight regain and associated mental health challenge (7).

Food addiction as a complex multifactorial eating behaviors disorder has high prevalence in obesity and may be improved by bariatric surgery (8, 9). According to a recent meta-analysis, the prevalence of food addiction in individuals with obesity could be as high as 32%, while between 2 and 12% of healthy individuals are affected by food addiction (10). Even adjusting for health factors (such as smoking, substance use and physical activity), food addiction still showed a significant association with obesity (11–13). A prospective study has shown that food addiction can be reduced by bariatric surgery, with prevalence rates decreasing from 57.8% preoperatively to 7.2 and 13.7% at the 6 and 12-month postoperative time points, respectively (14). It has also been observed clinically that in the early post-operative period after bariatric surgery (within 6 months), there is acute improvements in eating behaviors (15, 16). However, the situation of how food addiction continues to change after surgery has not been explicitly explored due to the lack of high temporal frequency and long cohort studies.

Food addiction can be characterized by a lack of control over food intake, strong appetite, excessive food consumption despite negative health or social consequences, and repeated unsuccessful attempts to control intake (17) and divided into eleven different symptoms, each of which describes an addictive behavior with different positivity rate by the Yale Food Addiction Scale 2.0 (YFAS 2.0) (18). Symptoms of food addiction are different, some are better recognized, such as behavioral signs, and some are not so well recognized, such as social relationships, which are often difficult to intervene in. A recent study has shown that the most endorsed symptom is the symptom ‘large amount / longer’ (19) as a common phenomenon among people with obesity (20), which was defined as substance taken in larger amount and for longer period than intended by YFAS 2.0. Its discrimination parameter remains high, indicating its relatively good ability to delineate individuals who are higher on the latent trait (19). In contrast, Mohsen Saffari considered ‘social/interpersonal problems’ as the most adopted symptom (21), which represented continued consumption despite social or interpersonal problems. While there was another result that the symptom ‘hazardous use’, which represented food use in physically hazardous situation, had a scarcity of endorsement and the lowest discrimination parameter indicating a poor ability to delineate individuals who are higher vs. lower on the latent trait (19). Consequently, the symptom ‘hazardous use’ may not be improved or improved slowly after people with obesity lose weight through bariatric surgery. There are also previous researches which mentioned that the ‘persistent desire or repeated unsuccessful attempts to quit’ symptom is the most commonly recognized criterion (22, 23). These studies have collectively concluded that self-reported food addiction has different rates of positivity for different symptoms. Although the identification of symptoms of food addiction varies among subjects, there is a lack of nooses regarding whether different symptoms of food addiction are equally improved by bariatric surgery. Even when the total food addiction score decreases after bariatric surgery but these symptoms with more recognized and represented do not improve well, people with obesity may still be suffering from food addiction.

Because the available evidence suggests that bariatric surgery reduces the incidence of postoperative food addiction, the symptoms associated with eating behaviors are more readily identifiable and ameliorated. Therefore, we hypothesized that: (1) bariatric surgery could rapidly alleviate postoperative food addiction symptoms in obese patients; (2) bariatric surgery is ineffective in alleviating some of the symptoms of interpersonal problems symptoms of food addiction.

## Methods

### Subjects

This study was part of a larger prospective cohort study at Shanghai Sixth People’s Hospital. We obtained the following baseline information before patients underwent bariatric surgery: age, years of education, sex, body mass index (BMI), the YFAS 2.0 (food addiction score). Based on the Chinese criteria for overweight and obesity, the overweight or obesity (OW/OB) group comprised patients with overweight or obesity (BMI > 26 kg/m2) who were undergoing bariatric surgery at the Weight Loss Metabolism Clinic in Shanghai Sixth People’s Hospital. The healthy control (HC) group comprised individuals with healthy BMIs (18 kg/m2 < BMI < 24 kg/m2) who were randomly recruited. Postoperative follow-up examinations were performed at 1-month intervals until 18 months postoperatively and then at 6-month intervals until 24 months postoperatively. All participants had to meet the following inclusion criteria: (i) age between 18 and 65 years, (ii) the capacity to give informed consent, (iii) no untreated mental illness or unstable mental state, (iv) all participants with a history of bariatric surgery, and pregnant or breast-feeding females were excluded. The study and the survey obtained ethics approval by the independent IRB of the authors’ institution (Ethics Committee of Shanghai Sixth People’s Hospital, NO 2020–219-(1)).

### Measurements

#### YFAS 2.0

The YFAS 2.0, which contained a total of 11 judgment criteria and one clinical diagnostic criterion, was revised and published in 2016 by Gearhardt et al. on this basis (18). The method of analysis was a questionnaire containing personal information and the YFAS 2.0 carried out in an online platform (www.wjx.cn). The YFAS 2.0 consists of 35 questions scored on an 8-level Likert scale ranging from 0 to 7. It provides two scoring methods: symptom count or diagnostic threshold. The presence of no more than one symptom or the absence of a symptom 12 was classified as non-addiction, the presence of 2–3 symptoms and the occurrence of a symptom 12 was classified as mild addiction, the presence of 4–5 symptoms together with the symptom 12 addiction was classified as moderate and the occurrence of more symptoms with 12 was classified as severe addiction. The Chinese version of the YFAS 2.0 had good internal consistency in our study. Cronbach’s *α* for the YFAS 2.0 was 0.93. For convenience, we abbreviated each symptom using one or few words. Detailed description of the symptoms can be found in Table 1.

**Table 1:** Cronbach’s *α* for each symptom the YFAS2.0.

Abbreviations	Subdimension (symptom)	Item-rest correlation	If item dropped Cronbach’s α
Large amount/longer	Substance taken in larger amount and for longer period than intended	0.656	0.925
Quit/control	Persistent desire or repeated unsuccessful attempts to quite	0.742	0.922
Time spent	Much time/activity to obtain, use, recover	0.733	0.922
Activities given up	Important social, occupational, or recreational activities given up or reduced	0.675	0.924
Psychological/physical problem	Use continues despite knowledge of adverse consequences (e.g., emotional problems, physical problems)	0.775	0.921
Tolerance	Tolerance (marked increase in amount; marked decrease in effect)	0.704	0.923
Withdrawal	Characteristic withdrawal symptoms; substance taken to relieve withdrawal	0.662	0.925
Social/interpersonal problems	Continued use despite social or interpersonal problems	0.579	0.929
Neglect role	Failure to fulfill major role obligation (e.g., work, school, home)	0.677	0.924
Hazardous use	Use in physically hazardous situations	0.671	0.925
Craving	Craving, or a strong desire or urge to use	0.762	0.922

### Statistical methods

All the statistical analyses were performed using Jamovi v2.3.16, GraphPad v 9.4.1 and R 4.2.3. The normally distributed data were analyzed by Student’s t-test and expressed as mean ± standard deviation. The non-normally distributed data were analyzed by the Mann–Whitney U test and expressed as median (quartiles [P25, P75]; the sex-related characteristics of the two groups were tested by the chi-square test).

For our primary endpoint, we conducted a mixed linear model (MLM) using food addiction score as the dependent variable. In this model, individual patients were included as a random effect, and the data timepoint for YFAS 2.0 collection as fixed effects. The MLM model was as follows:


Food addiction score~1+time+(1∣Participants)

Hierarchical clustering was performed using the Ward clustering algorithm and separated by Euclidean distance with factors arranged visually by time point and by groups. To assess the statistical significance of the clustering solution, we conducted an analysis of variance (ANOVA) to compare the means of variables across clusters. Our results indicated significant differences (*p* < 0.05) in the means of variables between clusters, supporting the validity of the clustering solution.

The model effectively controls for baseline heterogeneity between individuals by incorporating random intercept terms. It is suitable for longitudinal data analysis with repeated measurements. Model parameter estimation uses restricted maximum likelihood estimation (REML), and statistical significance is set at *α* = 0.05. Model fit was evaluated using the Akaike Information Criterion (AIC) and Bayesian Information Criterion (BIC). All statistical modeling procedures, including model fitting and validation, were implemented using dedicated open-source packages in R 4.4.3.

## Results

### Characteristics of the participants

Group comparisons of the sociodemographic characteristics; the BMI; and the food addiction score are shown in Table 2. The OW/OB group consisted of 78 subjects (61 females and 17 males) with a median age of 29.12 years. The HC group consisted of 65 subjects (51 females and 14 males) with a median age of 27.79 years. There were no significant differences in gender (*p* = 0.0970) or age (27.79 [25.27, 32.90] vs. 29.12 [24.53, 33.39], respectively; U = 2499.50, *p* = 0.886) between the two groups. The BMI and food addiction score in the HC group significantly differed from those in the OW/OB group before surgery; specifically, the OW/OB group had higher food addiction score. The bar chart showed that before surgery, the median food addiction score was significantly higher in the HC than OW/OB group (1 [0, 4] vs. 9 [4.25, 11], respectively; U = 288.00, *p* < 0.001). 34.6% of individuals in the OW/OB group received a diagnosis of food addiction, while 7.7% of individuals in the HC group received a diagnosis of food addiction.

**Table 2:** Demographics of all participants.

Characteristic	HC M (P25, P75)	OW/OB M (P25, P75)	Statistic	*p*	Effect size
Gender (M/F)	14/51	17/61	0 ^d^	0.970	/
Age (years)	27.79 (25.27, 32.90)	29.12 (24.53, 33.39)	2499.50	0.886	0.04
BMI (kg/m2)	20.97 ± 1.38 ^a^	38.25 ± 5.97 ^a^	−22.8 ^b^	< 0.001	−3.83^c^
Education years	16 (15, 18)	15 (12, 16)	33.02	0.007	0.32
FA score	1 (0, 4)	9 (4.25, 11)	288.00	< 0.001	0.734
Mild FA	1	1			
Moderate FA	2	1			
Severe FA	2	25			

HC, healthy control; OW/OB, overweight or obesity; M, Median; P25, 25th percentile; P75, 75th percentile; *p*, *p* value; Statistic is Mann–Whitney U test; Effect Size for Mann–Whitney U test is Rank biserial correlation. BMI conforms to the normality test, ^a^ mean ± Standard deviation, ^b^ Statistic is Student’s t, ^c^ Effect size for t test is Cohen’s d; Gender used chi-squared test, ^d^ statistic is *χ*^2^.

### Prevalence of food addiction categorization after SG

**Figure 1:**
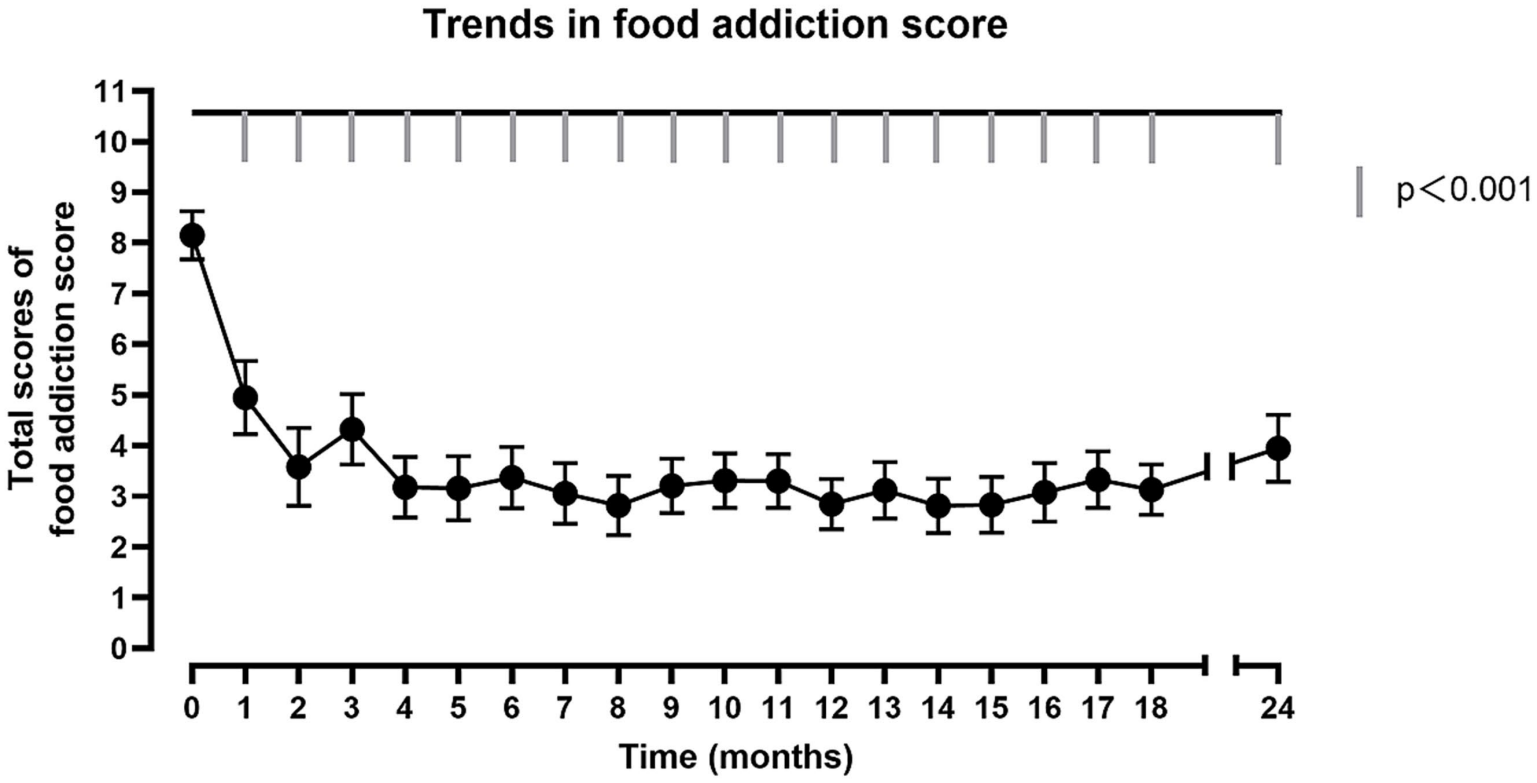
The trend of total food addiction score. The horizontal axis represents the post-operative follow-up time. The vertical axis represents the total score of food addiction score. The fold line reflects the trend in food addiction scores after surgery. Significant differences (*p* < 0.001) for each post-operative month compared to pre-operative.

To evaluate the assessment that SG could improve the situation of the food addiction, the MLM was used to describe the trend of the food addiction score, which took postoperative time as predictive variable, the food addiction score as predicted variable, subject as cluster. Compared with preoperative, the food addiction score of participants significantly decreased at 1 month postoperative (*t* = −4.64, *p* < 0.001), and it sustained until 24 months after bariatric surgery (*t* = −6.31, *p* < 0.001) (Figure 1; Supplementary Table S1). Finally, 25% of individuals in the OW/OB group received a diagnosis of food addiction at 24 months postoperative. In addition to the preoperative comparisons, repeated comparisons were made, i.e., changes were compared between two adjacent time points to determine at which postoperative time point the participants’ food addiction improvement occurred primarily. It was found that food addiction scores only decreased significantly in the first month postoperatively compared to the preoperative period, and otherwise did not change significantly compared to the previous month (Supplementary Table S2).

Analysis of standardized residuals from the model indicated approximate normality in the overall error distribution. The residual median was −0.0729, demonstrating near-symmetry about zero. Interquartile ranges spanned from −0.454 (lower quartile) to 0.409 (upper quartile), satisfying the normality assumption for linear model errors. Extreme values ranged from −3.379 to 3.891, suggesting potential outliers. The model was estimated using restricted maximum likelihood (REML), with convergence achieved at an objective function value of 2,207. Model fit indices yielded an AIC of 2250.97 and a BIC of 2343.02.

### Response of each symptom in YFAS 2.0 after SG

**Figure 2:**
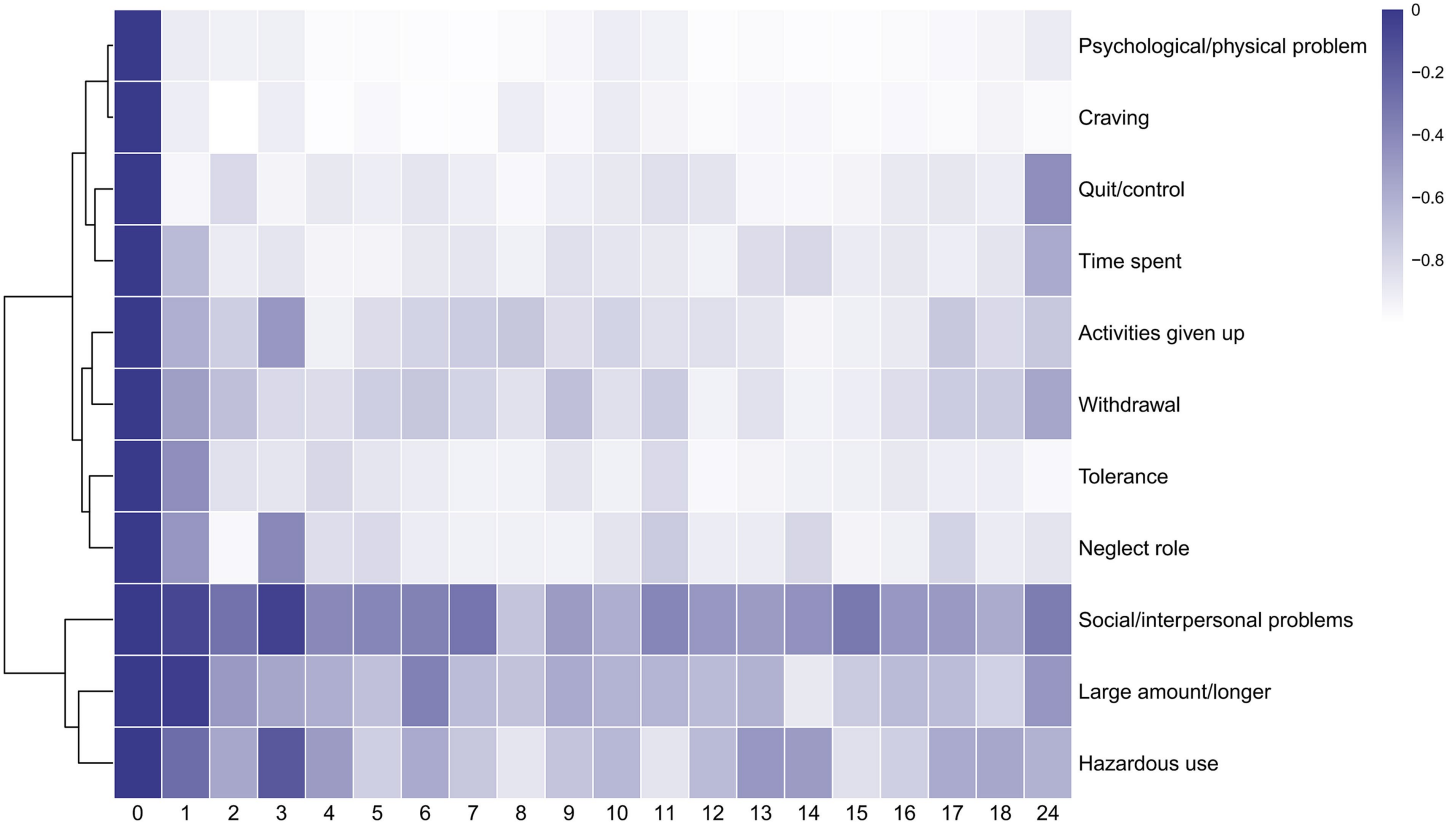
Heatmap of 11 symptoms of YFAS 2.0. The horizontal axis represents the post-operative follow-up time. The vertical axis represents 11 symptoms of YFAS 2.0(‘Large amount/longer’ for substance taken in larger amount and for longer period than intended. ‘Quit/control’ for persistent desire or repeated unsuccessful attempts to quit. ‘Time spent’ for much time/activity to obtain, use, recover. ‘Activities given up’ for important social, occupational, or recreational activities given up or reduced. ‘Psychological/physical problem’ for use continues despite knowledge of adverse consequences. ‘Tolerance’ for tolerance. ‘Withdrawal’ for characteristic withdrawal symptoms; substance taken to relieve withdrawal. ‘Social/interpersonal problems’ for continued use despite social or interpersonal problems. ‘Neglect role’ for failure to fulfill major role obligation. ‘Hazardous use’ for use in physically hazardous situations. ‘Craving’ for craving, or a strong desire or urge to use.) The algorithm for heatmap clustering was based on the Euclidean distance measure for similarity and Ward clustering algorithm.

In order to observe how the proportion of positive individuals for each symptom in the YFAS 2.0 description varied, a cluster heat map was created based on each participant’s response to the questionnaire. In the heat map, each row corresponds to a symptom in the YFAS 2.0 description, whereas the columns represent the various pre- and post-operative time points. The values represented by each square are calculated according to a generalized MLM. Increasing brightness toward blue indicates a higher mean of possible rate, and white indicates a lower mean of possible rate. We found that out of a total of 11 symptoms, ‘psychological/ physical problem’, ‘craving’, ‘quit/control’, ‘time spent’, ‘activities given up’, ‘withdrawal’, ‘tolerance’, and ‘neglect role’ showed more similar trends, ‘social/interpersonal problems’, ‘large amount/longer’, and ‘hazardous use’ showed more similar trends (Figure 2).

In addition, the post-operative trends for each symptom were represented by equations calculated from a generalized MLM and presented as line graphs. For specific trends in each symptom, see Fig. S1 - Fig S11 in the Supporting Materials. We found that not all symptoms began to improve in the early postoperative period immediately. Among the symptoms ‘large amount/longer’ (*z* = −0.243, *p* > 0.05), ‘tolerance’ (*z* = −1.10, *p* > 0.05), ‘social/interpersonal problems’ (*z* = −1.27, *p* > 0.05), ‘hazardous use’ (*z* = −1.116, *p* > 0.05), there was no significant difference in the first month postoperatively compared to preoperatively. The symptom ‘large amount/longer’ began to improve significantly at the second month postoperatively (*z* = −2.571, *p* < 0.05). The symptom ‘hazardous use’ was improved significantly at the second month postoperatively (*z* = −2.039, *p* < 0.05) compared to the preoperative, while did not differ at the third month postoperatively (*z* = −0.719, *p* > 0.05) compared to the preoperative. The symptom ‘social/interpersonal problems’ was improved significantly at the second month postoperatively (*z* = −2.50, *p* < 0.05) compared to the preoperative, while did not differ at the third month postoperatively (*z* = −1.21, *p* > 0.05) compared to the preoperative. The symptom ‘hazardous use’ and ‘social/interpersonal problems’ were both improved from 4th month postoperatively until 24 months after bariatric surgery.

## Discussion

This study sought to explore the interventional effects of bariatric surgery on food addiction by continuous measurements at intensive postoperative time points. Two main new findings were found: 1. Total food addiction scores were substantially reduced by bariatric surgery within 1 month postoperatively, and it could be maintained for at least 24 months postoperatively; 2. Bariatric surgery improved most of the symptoms of food addiction in the early postoperative period, however, it was slower to improve slow decline domain.

### Bariatric surgery improves food addiction scores

Food addiction scores started to decline already early after bariatric surgery and leveled off afterwards. This study found that 34.6% of candidates for bariatric surgery have suffered from food addiction and exhibited elevated addiction scores, aligning with another observation reported by our research team (24). And it declined to 25% 2 years postoperatively. This is in line with the results of a recent meta-analysis shown that the absolute prevalence reduction of food addiction was decreased after bariatric surgery. Further, the effect was observed within 6 months of postoperative follow-up (10). We speculate that this may be due to neurophysiology, gut microbiota changes and eating behavior factors in the patients as a result of the bariatric surgery.

Food addiction is largely reduced due to a restriction of the gastric volume after bariatric surgical intervention, during which neuromodulation and hormone regulation are stimulated. The addiction is mediated by brain regions and neurotransmitters (25). Bariatric surgery reduces appetite and induces psychophysiological effects by influencing the expression of numerous brain neurotransmitters, including dopamine, serotonin and various neuropeptides such as neuropeptide Y, leptin, orexin, growth hormone-releasing peptide, growth hormone-releasing factor, leptin and glucagon-like peptide-1 levels (26). Bariatric surgery alters these neuropeptides’ secretion, which in turn reduces the patients’ food craving and lack of control over food intake (27, 28), important addiction symptoms in food addiction.

Bariatric surgery affects food addiction by altering a patient’s gut microbiota, which may in turn affect food addiction. Environmental factors and dietary patterns have a major influence on gut microbiota composition, and the overconsumption of highly palatable food may promote a gut microbiota dysbiosis that has been recently proposed to participate in the loss of eating control (29). Individuals with obesity, which may be promoted by food addiction, showed altered gut microbiota with a reduced diversity that facilitated energy absorption capacity and may affect host brain function (30). In the recent study, researchers demonstrated a translational link between mice and humans in gut microbiome composition associated with food addiction, supporting a link between gut microbiota and vulnerability to this behavioral disorder (31). While bariatric surgery affects the hosts’ metabolism by altering the gut microbiota (32) and modulates the inflammatory response through a variety of mechanisms that alter the patient’s physiology (33), which is a potential way to improve food addiction.

Food addiction is clinically associated with dysregulated eating patterns including grazing (34), emotional eating, and loss-of-control (LOC) eating (35). Grazing, operationally defined as the persistent, compulsive consumption of small-to-moderate food volumes without discrete meals or temporal structure (36), constitutes a distinct yet potentially mutually reinforcing behavioral phenotype relative to food addiction. Symptomatic overlap exists between grazing and food addiction (34), postoperative grazing may attenuate weight loss efficacy following bariatric surgery (37). Surgical gastric restriction necessitates structured nutritional protocols; consequently, postoperative guidance advocates planned, repetitive meal patterns with prescribed dietary composition. Some authorities contend that intention-regulated eating should be nosologically distinguished from maladaptive grazing (36). Some evidences suggests grazing incidence may increase postoperatively despite these interventions (37, 38). This investigation observed sustained reductions in food addiction scores across a 24-month postoperative follow-up. Crucially, this amelioration persisted even when accounting for putative risk factors associated with elevated postoperative grazing prevalence. A recent study proposed a cyclical reinforcement model wherein emotional eating drives dysregulated consumption, precipitating loss-of-control eating (35). Subsequent maladaptive attempts to regulate this behavior paradoxically perpetuate and intensify food addiction. This model emphasizes the mediating role of food-related cognitions (e.g., food craving) in facilitating LOC episodes, establishing cognitive processes as critical mechanisms in food addiction. Studies indicate bariatric surgery significantly attenuates emotional and LOC eating behaviors while enhancing cognitive restraint in candidates (39, 40). This suggests surgical intervention may ameliorate food addiction symptomatology through modulation of these core behavioral pathways. Notably, a meta-analysis revealed cognitive behavioral therapy (CBT) produces significant short-term reductions in emotional eating post-intervention (41). However, these gains demonstrate poor sustainability at long-term follow-up. Critically, CBT showed no significant effects on sustained LOC improvement at long-term follow-up. These findings remain constrained by limited sample sizes and methodological heterogeneity, precluding definitive conclusions regarding CBT’s long-term efficacy. This study documented postoperative food addiction score reduction absent concurrent CBT implementation.

Thus, the data from this study suggest that symptoms of food addiction in patients with obesity can be ameliorated by bariatric surgery. However, previous research on of postoperative outcomes in early food addiction has predominantly focused on follow-up assessments conducted beyond 3 months (42, 43). Although some studies have employed short-term follow-up at 1 month, sequential monthly assessments are notably lacking (44). More and longer longitudinal studies, preferably with experimental designs with control groups, are needed to assess the impact of bariatric surgery on the negative effects of food addiction. In addition, we should further investigate the mechanisms to provide a theoretical basis for finding new treatment pathways for food addiction in the future. As for the patients, we can better manage obesity in this population and reduce the symptoms of food addiction in patients.

### Slow improvement in symptom ‘social/interpersonal problems’, ‘hazardous use’ and ‘large amount/longer’

All eleven symptoms can be classified into two domains based on postoperative trends, with the first domain improving rapidly postoperatively and the second domain improving slowly postoperatively. The rapid decline domain includes ‘psychological/ physical problem’, ‘craving’, ‘quit/control’, ‘time spent’, ‘activities given up’, ‘withdrawal’, ‘tolerance’, and ‘neglect role’; the slow decline domain includes ‘social/interpersonal problems’, ‘large amount/longer’, and ‘hazardous use’. The slowest improvement after surgery is seen in ‘social/interpersonal problems’.

Subjects are sensitive to the social and psychological problems caused by food addiction. The highest number of subjects (40/46) suffered from this problem before surgery, and the tendency to decline after surgery was moderate compared to other symptoms. It was obvious that the symptom for persistent desire or repeated unsuccessful attempts to quit was reduced by bariatric surgery rapidly in the early post-operative period. No previous study has followed up so intensively after surgery and analyzed serial changes in different symptoms. Patients involved in bariatric surgery are obese, a group of patients who face physical impairment, mobility difficulties, low self-esteem or self-control, and illnesses associated with negative body imagery (45, 46). Women may also be more concerned about aesthetic issues and may refuse and not want to disclose their weight. Whereas patients with obesity are often unable to perceive their weight correctly, even after bariatric surgery, patients seem unable to recognize changes in body image following significant weight loss (45). This can lead to patients refusing to participate in social activities for fear of overeating in front of their loved ones or friends, even though they have lost weight after bariatric surgery. This also explains why ‘social/interpersonal problems’ in the YFAS 2.0 is relatively slow to improve after surgery. A better understanding of this unique postoperative symptom may help to elucidate the underlying biological and psychological bases associated with these more severe symptoms, especially after bariatric surgery.

‘Hazardous use’ symptom is slow to improve and tend to rebound after bariatric surgery. ‘Hazardous use’ is the behavior of eating certain foods even when individuals know it was physically dangerous (such as eating sweets even with diabetes), which is one of the main symptoms of food addiction. Some studies have found that over time, cravings for sweets no longer differ from pre-operative (47). We advise patients to eat more foods rich in high protein postoperatively, and sweets such as sugar and beverages are strongly discouraged foods because eating sweets can cause patients to regain weight, as well as being detrimental to diabetes and insulin resistance controlling. This may lead patients to feel that they are hurting themselves by eating sweets, but when they still insist on eating sweets, they self-report as hazardous use. In addition, a recent finding suggests that some patients may substitute the concept of one reinforcer ‘food’ for another, such as ‘alcohol’, which is commonly referred to as ‘addiction transfer’ (48). And these patients with addiction shifts can have alcohol abuse, which may be contributing to the slow decline in symptom of ‘hazardous use’ after patients undergo bariatric surgery, despite a rapid decline in food addiction scores.

‘Large amount/longer’ begins to decline in the second month and remains at a lower level from the second month to the 24th month postoperatively. ‘Large amount/longer’ describes eating behaviors in which individuals eat more than planned or for more time than planned, persist in eating in the absence of hunger and eat to the point where they fell physically ill. Previous studies have shown that patients’ hunger, disinhibition and emotional eating decrease postoperatively, whereas restraint and postprandial fullness increase (49, 50). This may lead to an improvement in ‘large amount/longer’ postoperatively, which is in line with the trend we observed for postoperative changes. Besides, the new finding is that the improvement of ‘large amount/longer’ starts in the second postoperative month. We hypothesize that the unimprovement of the first month may be caused by the following reasons. Firstly, overall maladaptive eating behaviors (binge eating, night eating and uncontrolled eating) which are associated with ‘large amount/longer’ improve postoperatively and require an adaptation process (51). Secondly, the recovery of the postoperative diet starts with a liquid diet and gradually transitions to a semi-liquid, soft food and then to a normal diet. We commonly instruct the patients to eat slowly during the postoperative mission and to have an intermediate cut between ingesting solids and liquids at each meal, all of which may lead to an increase in the patient’s eating time. Thirdly, bariatric surgery alters the volume of the stomach, causing patients to feel fuller after surgery. In the early postoperative period, eating too quickly or eating too much at once may cause the patients to feel physically uncomfortable. These may explain why ‘large amount/longer’ did not improve significantly in the first month after surgery compared to the preoperative.

The following are a few limitations of this study. Prior research lacks studies utilizing MLM to characterize the continuous postoperative trajectory of food addiction scores. Consequently, no established reference exists to inform power calculations or sample size estimation for this specific analytical approach. Our sample size was therefore informed provisionally by effect sizes reported in studies examining short- and long-term food addiction improvement following bariatric surgery, with an anticipated attrition rate of 20% incorporated. During longitudinal assessment, attrition rates exceeded the projected 20% threshold at several follow-up intervals. The implementation of MLM for trajectory analysis— rather than reliance on mean food addiction score reporting— partially mitigates the impact of this missing data by accommodating subject-wise inclusion of all available observations. Consequently, despite acknowledged limitations in cohort retention, the reported model outputs remain methodologically robust and clinically interpretable. Future investigations should incorporate longitudinal assessments of dysregulated eating behaviors, trait-level food cravings, and dynamic psychological states. Integrating these multidimensional covariates would refine model specification accuracy and elucidate mechanistic pathways through which bariatric surgery ameliorates food addiction. This study was more of a questionnaire and a behavioral experiment to collect information about the patients, and more biochemical and neuroimaging indicators are needed to help us explain the mechanisms behind this phenomenon. Some patients may have had inadequate energy or time to complete the questionnaire on schedule, instead completing it a few days later than planned. All of the surgeries were performed using sleeve gastrectomy, and we can use the type of surgery as a controlled variable in the future study.

## Conclusion

Bariatric surgery induced rapid and clinically meaningful improvement in food addiction symptomatology, with significant reductions observed as early as 1 month postoperatively. This therapeutic effect persisted throughout the 24-month follow-up period. However, the rates of improvement manifested significant heterogeneity across symptom domains. Notably, amelioration progressed more gradually for items assessing ‘Social/interpersonal problems’, ‘Hazardous use’, and ‘Large amount/longer’ compared to other symptoms.

## Data Availability

The data supporting the conclusions of this article will be made available by the authors upon request.

## Supplementary material

SUPPLEMENTARY FIGURES S1-S11 Trends in each symptom of the YFAS 2.0.
