# Supplementary material for: When bariatric surgery reduces food addiction: a prospective study
Source: Front Nutr. 2025 Nov 27;12:1535911. doi: 10.3389/fnut.2025.1535911 (PMC12695559; doi:10.3389/fnut.2025.1535911)
Supplement: SUPPLEMENTARY FIGURES S1-S11 — Trends in each symptom of the YFAS 2.0. [file Data_Sheet_1.zip › Supplementary Table 1.docx]

**Supplementary table**

**Table S1. The alterations of food addiction scores after surgery**

|  |  |  |  | **95% Confidence Interval** | |  |  |  |
| --- | --- | --- | --- | --- | --- | --- | --- | --- |
| **Names** | **Effect** | **Estimate** | **SE** | **Lower** | **Upper** | **df** | **t** | **p** |
| **(Intercept)** | (Intercept) | 3.67 | 0.393 | 2.90 | 4.44 | 68.4 | 9.34 | < .001 |
| **time1** | 1 - 0 | -3.12 | 0.694 | -4.48 | -1.76 | 400.5 | -4.49 | < .001 |
| **time2** | 2 - 0 | -4.47 | 0.745 | -5.93 | -3.01 | 398.5 | -6.00 | < .001 |
| **time3** | 3 - 0 | -3.71 | 0.668 | -5.02 | -2.40 | 399.4 | -5.56 | < .001 |
| **time4** | 4 - 0 | -4.87 | 0.568 | -5.98 | -3.75 | 402.1 | -8.57 | < .001 |
| **time5** | 5 - 0 | -4.79 | 0.615 | -6.00 | -3.59 | 400.3 | -7.79 | < .001 |
| **time6** | 6 - 0 | -4.64 | 0.568 | -5.76 | -3.53 | 402.3 | -8.17 | < .001 |
| **time7** | 7 - 0 | -4.99 | 0.570 | -6.10 | -3.87 | 402.8 | -8.75 | < .001 |
| **time8** | 8 - 0 | -5.23 | 0.549 | -6.30 | -4.15 | 402.8 | -9.52 | < .001 |
| **time9** | 9 - 0 | -4.85 | 0.516 | -5.87 | -3.84 | 407.8 | -9.41 | < .001 |
| **time10** | 10 - 0 | -4.77 | 0.518 | -5.79 | -3.76 | 413.9 | -9.21 | < .001 |
| **time11** | 11 - 0 | -4.77 | 0.514 | -5.78 | -3.77 | 415.6 | -9.29 | < .001 |
| **time12** | 12 - 0 | -5.28 | 0.478 | -6.22 | -4.34 | 415.2 | -11.04 | < .001 |
| **time13** | 13 - 0 | -5.00 | 0.558 | -6.10 | -3.91 | 409.9 | -8.96 | < .001 |
| **time14** | 14 - 0 | -5.31 | 0.529 | -6.35 | -4.28 | 410.7 | -10.05 | < .001 |
| **time15** | 15 - 0 | -5.25 | 0.530 | -6.29 | -4.21 | 408.6 | -9.91 | < .001 |
| **time16** | 16 - 0 | -5.01 | 0.561 | -6.11 | -3.91 | 407.1 | -8.94 | < .001 |
| **time17** | 17 - 0 | -4.76 | 0.539 | -5.81 | -3.70 | 408.6 | -8.83 | < .001 |
| **time18** | 18 - 0 | -4.96 | 0.487 | -5.91 | -4.00 | 420.9 | -10.19 | < .001 |
| **time19** | 24 - 0 | -3.99 | 0.726 | -5.41 | -2.57 | 427.5 | -5.49 | < .001 |

**Table S2. The alterations of food addiction scores after surgery (repeated comparison)**

|  |  | |  | |  | | **95% Confidence Interval** | |  |  |  |
| --- | --- | --- | --- | --- | --- | --- | --- | --- | --- | --- | --- |
| **Names** | | **Effect** | | **Estimate** | | **SE** | **Lower** | **Upper** | **df** | **t** | **p** |
| **(Intercept)** | | (Intercept) | | 3.67 | | 0.393 | 2.90 | 4.44 | 68.4 | 9.34 | < .001 |
| **time1** | | 0 - 1 | | 3.12 | | 0.694 | 1.76 | 4.48 | 400.5 | 4.49 | < .001 |
| **time2** | | 1 - 2 | | 1.35 | | 0.897 | -0.41 | 3.11 | 392.2 | 1.51 | 0.133 |
| **time3** | | 2 - 3 | | -0.76 | | 0.870 | -2.46 | 0.95 | 387.5 | -0.87 | 0.385 |
| **time4** | | 3 - 4 | | 1.16 | | 0.738 | -0.29 | 2.60 | 388.9 | 1.57 | 0.118 |
| **time5** | | 4 - 5 | | -0.07 | | 0.686 | -1.42 | 1.27 | 386.7 | -0.11 | 0.914 |
| **time6** | | 5 - 6 | | -0.15 | | 0.686 | -1.50 | 1.19 | 386.7 | -0.22 | 0.824 |
| **time7** | | 6 - 7 | | 0.35 | | 0.646 | -0.92 | 1.61 | 386.7 | 0.54 | 0.592 |
| **time8** | | 7 - 8 | | 0.24 | | 0.639 | -1.01 | 1.49 | 388.5 | 0.37 | 0.708 |
| **time9** | | 8 - 9 | | -0.37 | | 0.597 | -1.54 | 0.80 | 391.1 | -0.62 | 0.533 |
| **time10** | | 9 - 10 | | -0.08 | | 0.555 | -1.17 | 1.00 | 394.0 | -0.15 | 0.881 |
| **time11** | | 10 - 11 | | <0.01 | | 0.551 | -1.08 | 1.08 | 397.9 | 0.01 | 0.994 |
| **time12** | | 11 - 12 | | 0.51 | | 0.510 | -0.50 | 1.51 | 395.5 | 0.99 | 0.323 |
| **time13** | | 12 - 13 | | -0.28 | | 0.551 | -1.36 | 0.80 | 391.5 | -0.50 | 0.616 |
| **time14** | | 13 - 14 | | 0.31 | | 0.589 | -0.85 | 1.47 | 389.4 | 0.53 | 0.599 |
| **time15** | | 14 - 15 | | -0.06 | | 0.574 | -1.18 | 1.06 | 390.3 | -0.10 | 0.917 |
| **time16** | | 15 - 16 | | -0.24 | | 0.593 | -1.41 | 0.92 | 386.9 | -0.41 | 0.682 |
| **time17** | | 16 - 17 | | -0.25 | | 0.599 | -1.43 | 0.92 | 386.8 | -0.42 | 0.673 |
| **time18** | | 17 - 18 | | 0.20 | | 0.540 | -0.86 | 1.26 | 394.2 | 0.37 | 0.713 |
| **time19** | | 18 - 24 | | -0.97 | | 0.703 | -2.35 | 0.41 | 412.0 | -1.38 | 0.169 |
