# Supplementary figures and images for: When bariatric surgery reduces food addiction: a prospective study
Source: Front Nutr. 2025 Nov 27;12:1535911. doi: 10.3389/fnut.2025.1535911 (PMC12695559; doi:10.3389/fnut.2025.1535911)

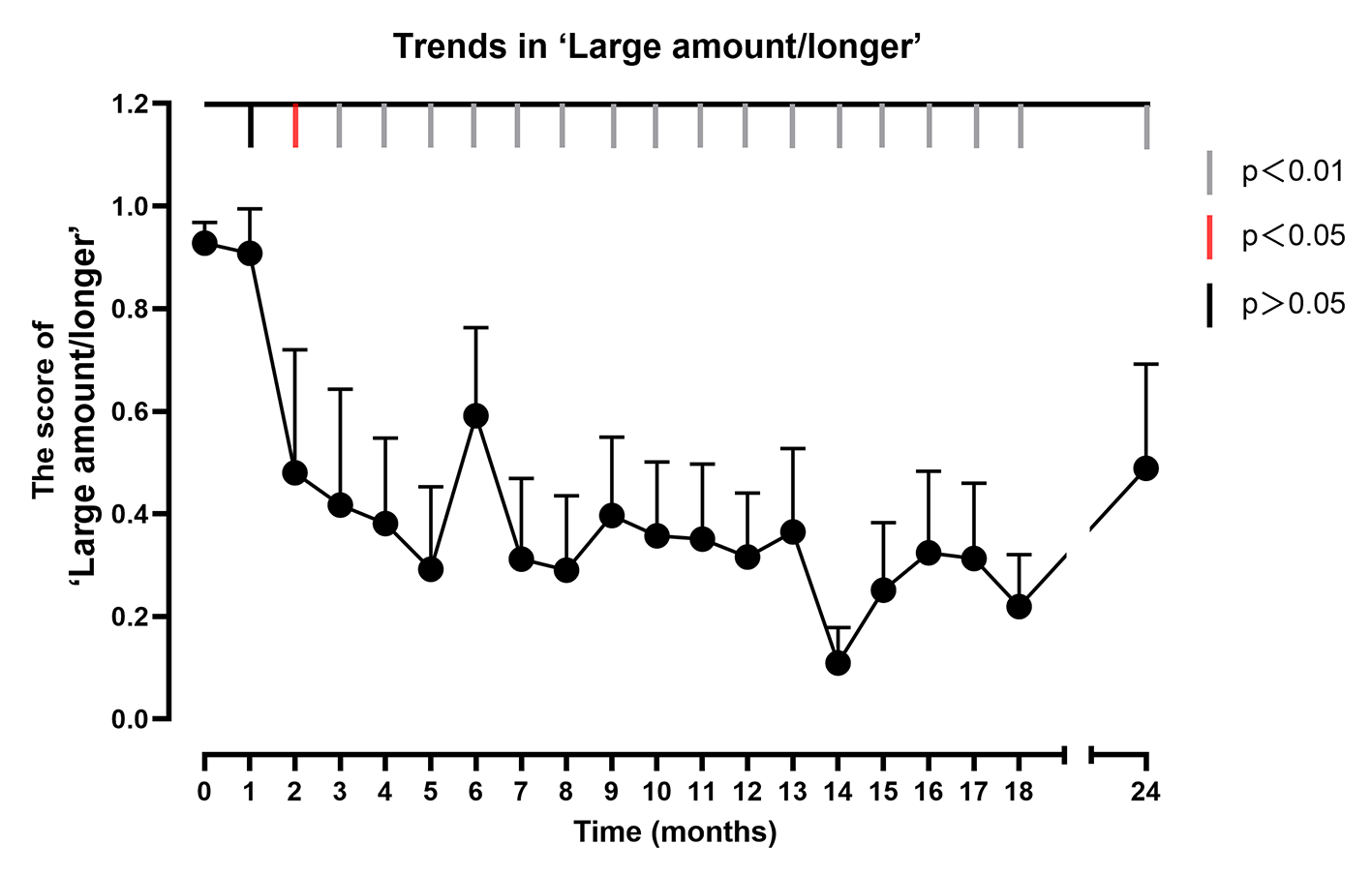

Supplement: SUPPLEMENTARY FIGURES S1-S11 — Trends in each symptom of the YFAS 2.0. [file Data_Sheet_1.zip › Image 1.TIF]

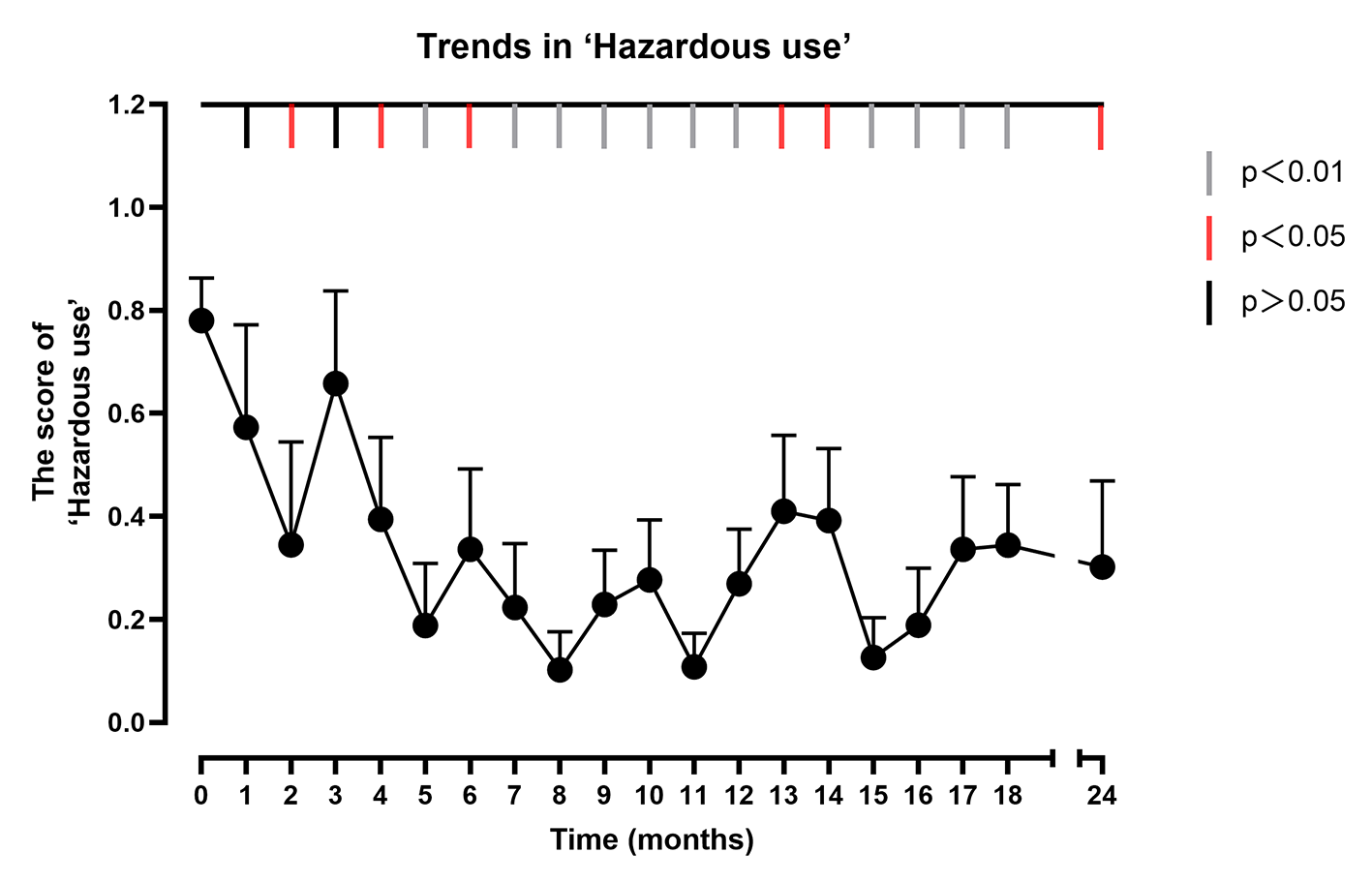

Supplement: SUPPLEMENTARY FIGURES S1-S11 — Trends in each symptom of the YFAS 2.0. [file Data_Sheet_1.zip › Image 10.TIF]

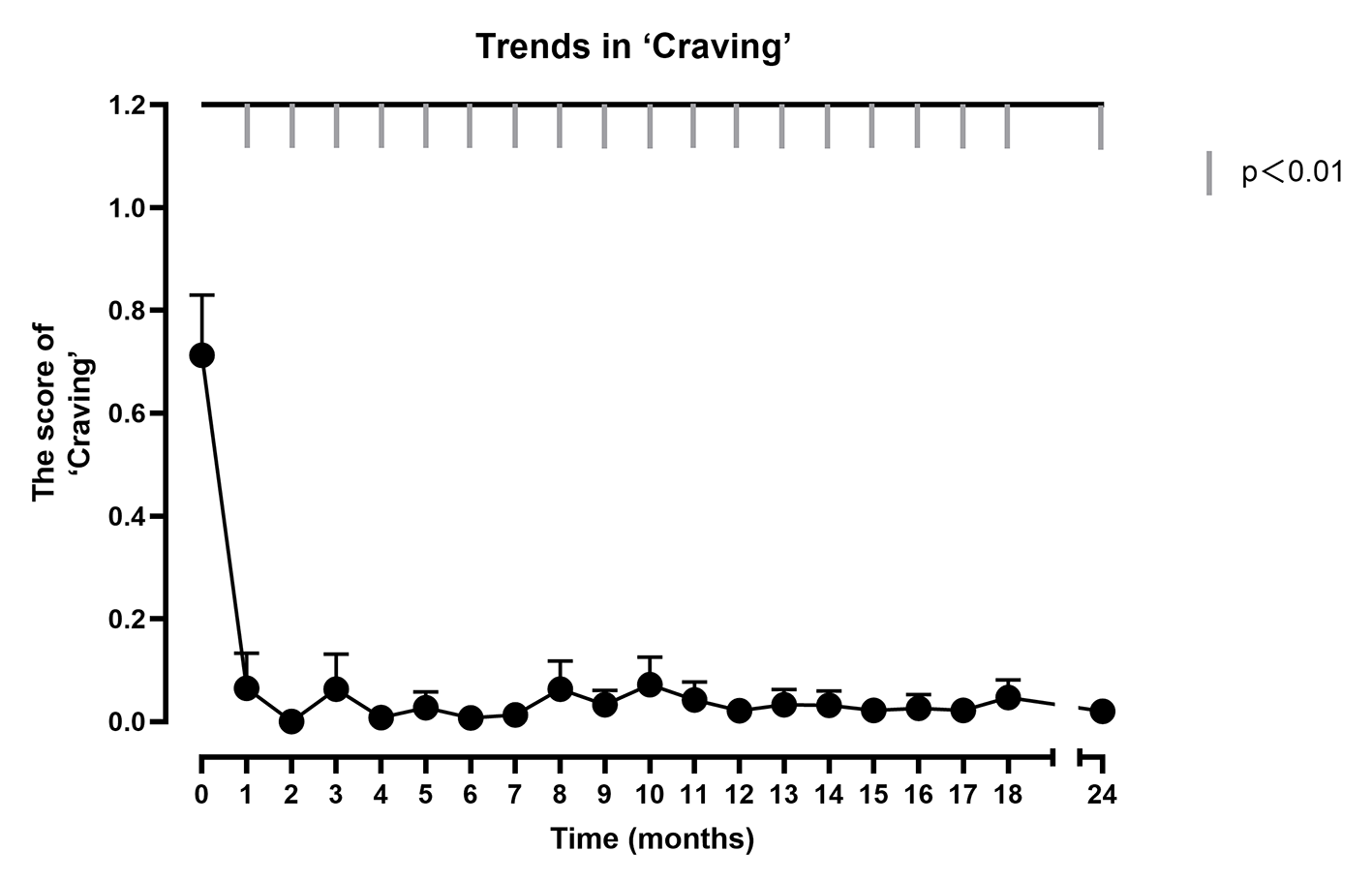

Supplement: SUPPLEMENTARY FIGURES S1-S11 — Trends in each symptom of the YFAS 2.0. [file Data_Sheet_1.zip › Image 11.TIF]

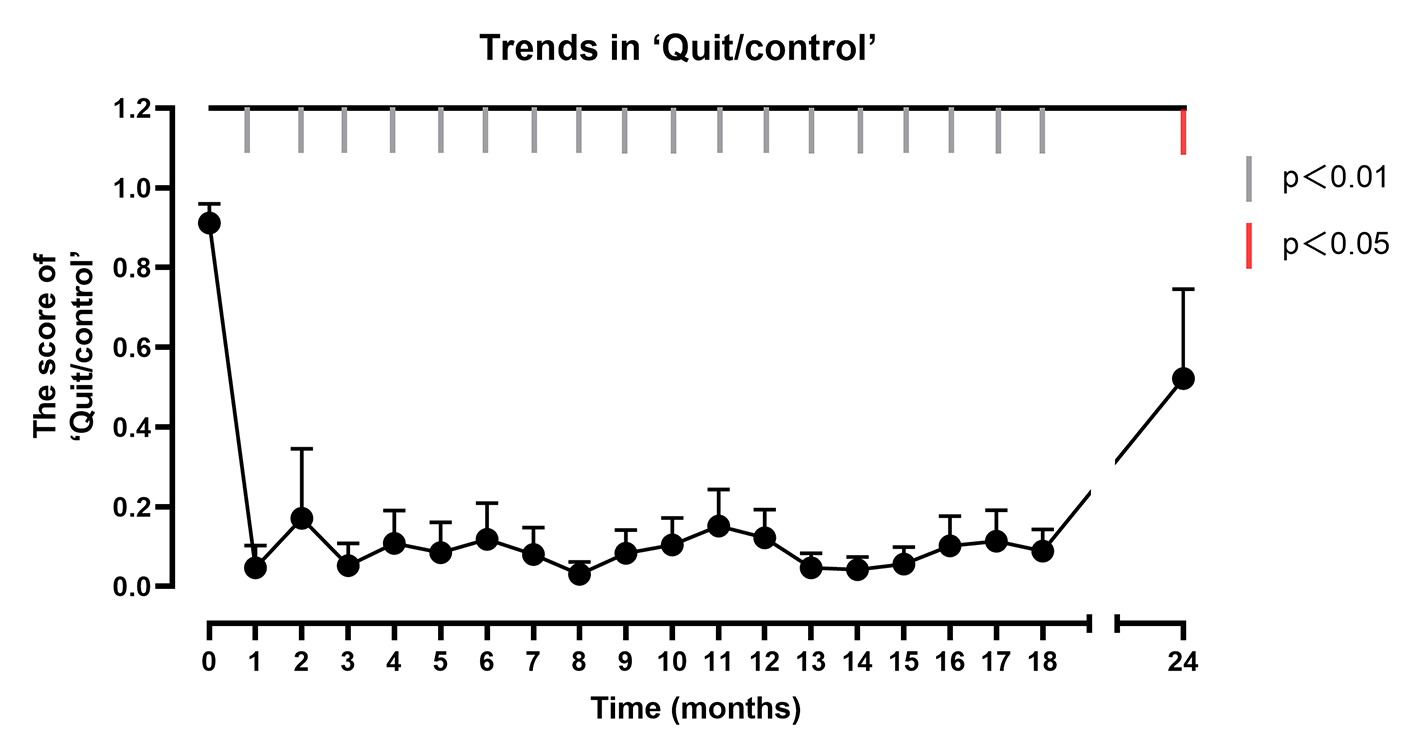

Supplement: SUPPLEMENTARY FIGURES S1-S11 — Trends in each symptom of the YFAS 2.0. [file Data_Sheet_1.zip › Image 2.TIF]

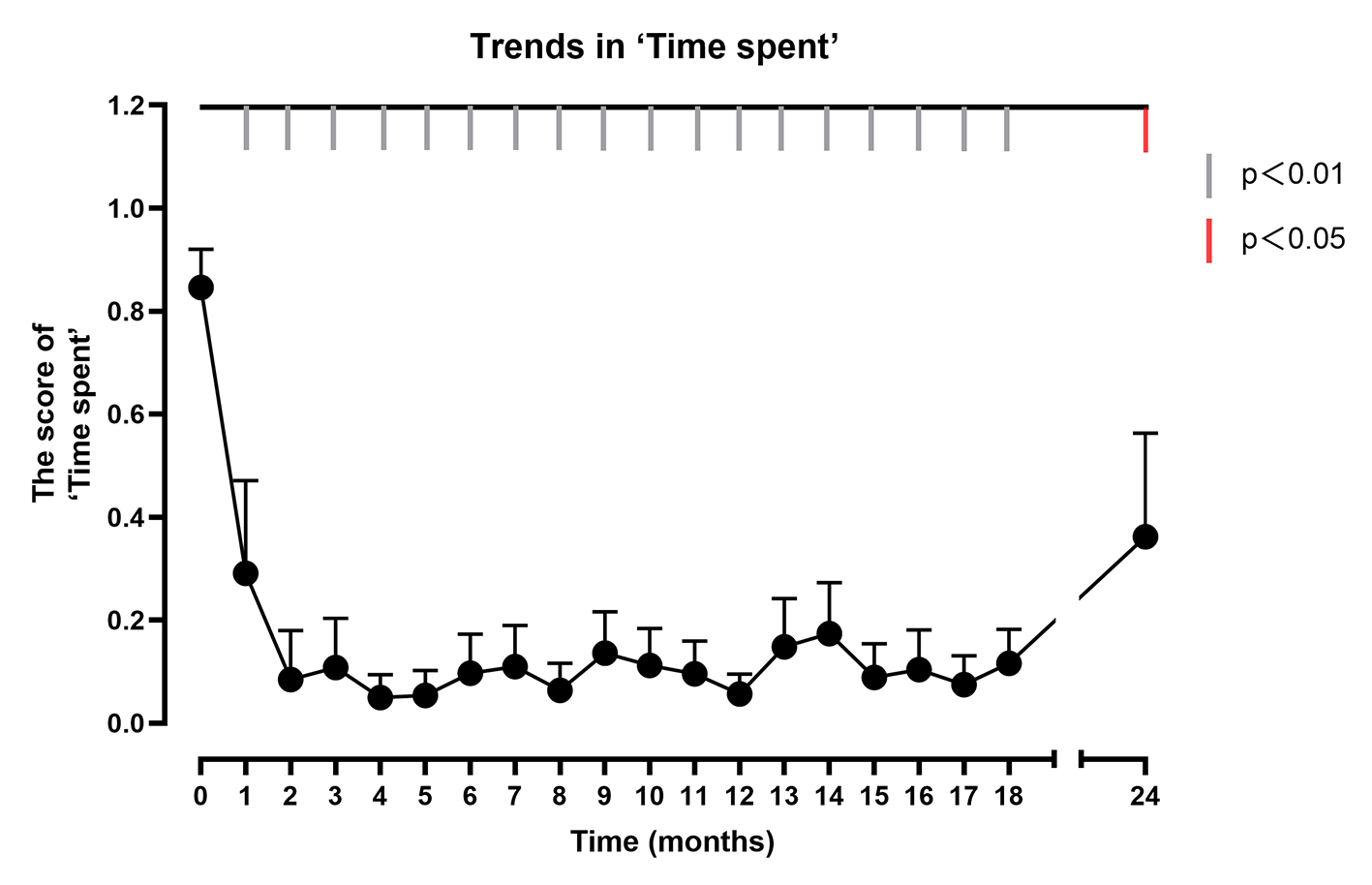

Supplement: SUPPLEMENTARY FIGURES S1-S11 — Trends in each symptom of the YFAS 2.0. [file Data_Sheet_1.zip › Image 3.TIF]

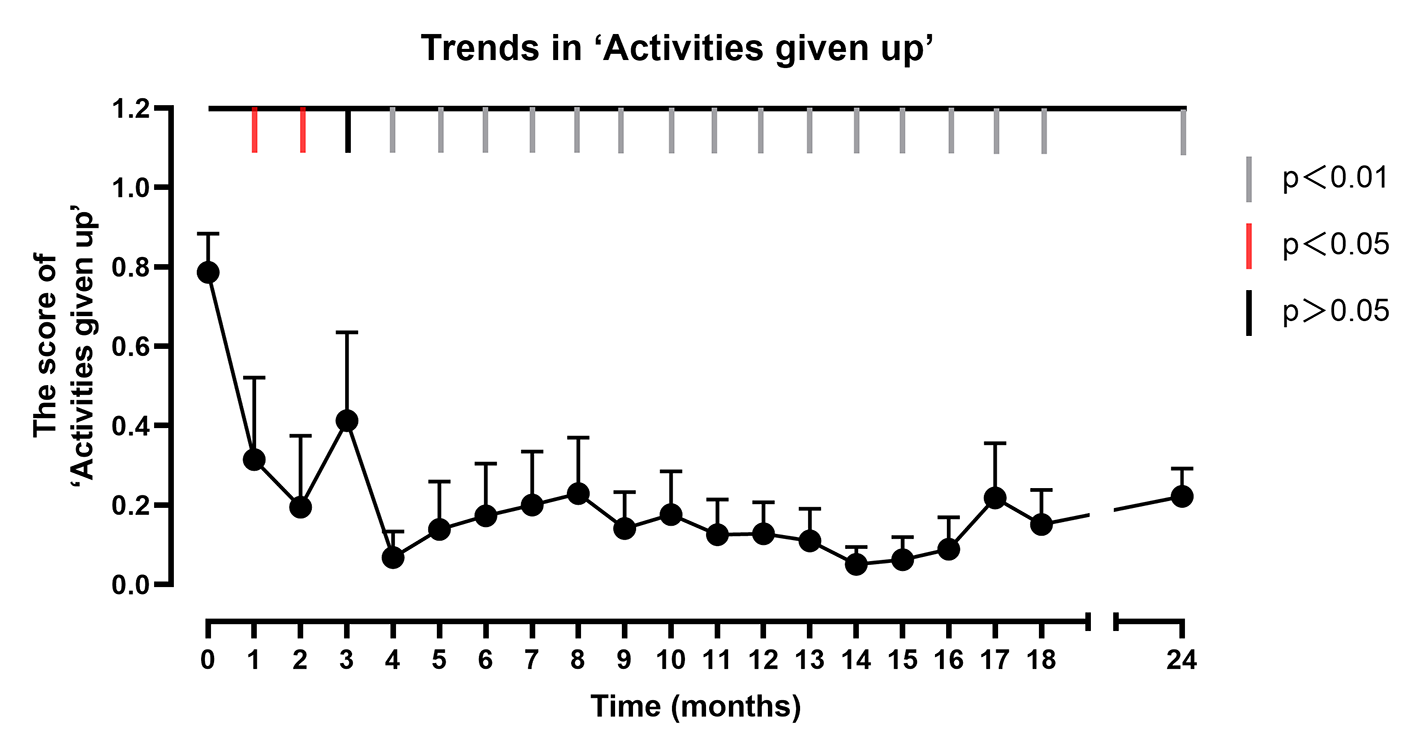

Supplement: SUPPLEMENTARY FIGURES S1-S11 — Trends in each symptom of the YFAS 2.0. [file Data_Sheet_1.zip › Image 4.TIF]

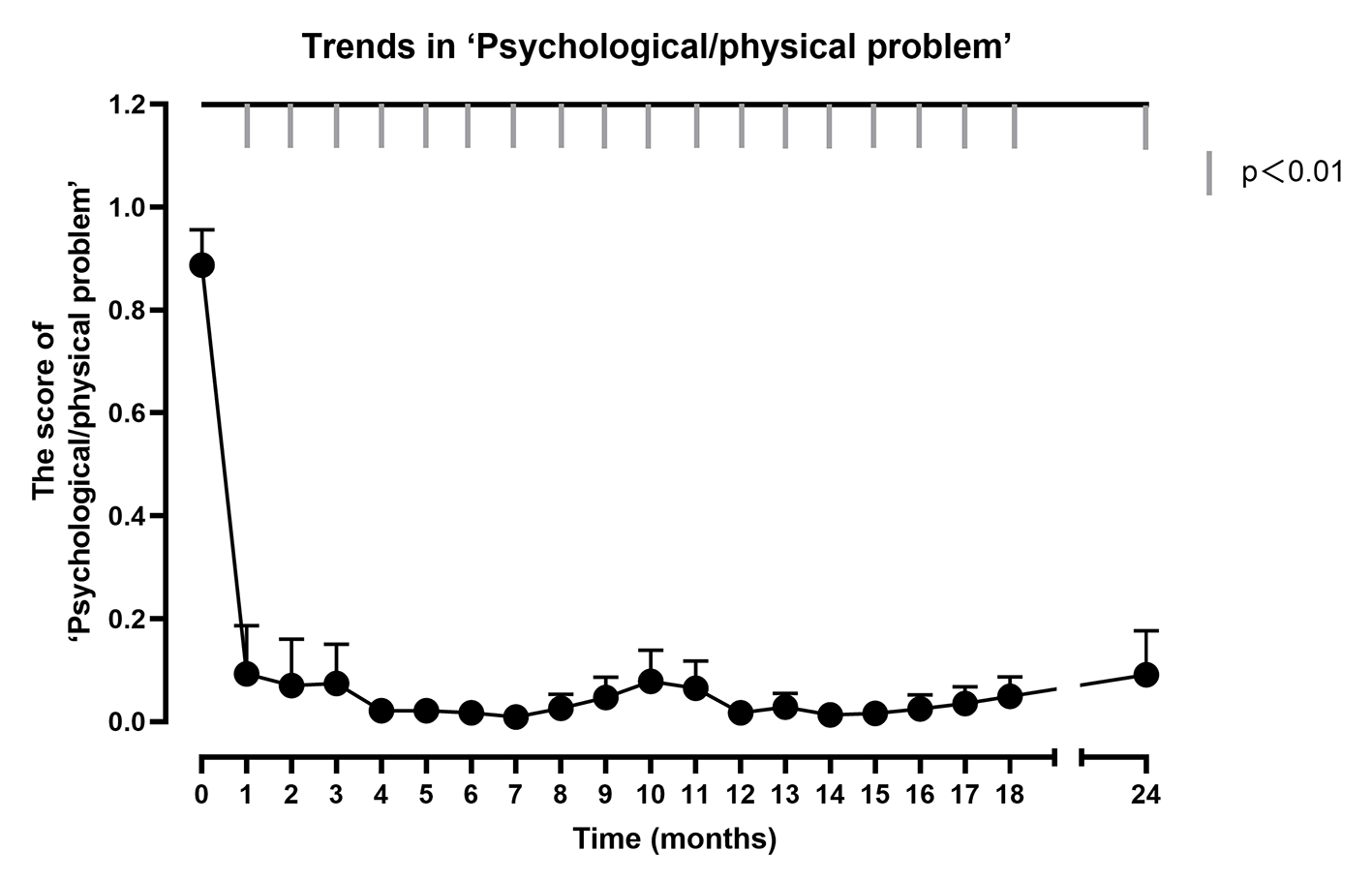

Supplement: SUPPLEMENTARY FIGURES S1-S11 — Trends in each symptom of the YFAS 2.0. [file Data_Sheet_1.zip › Image 5.TIF]

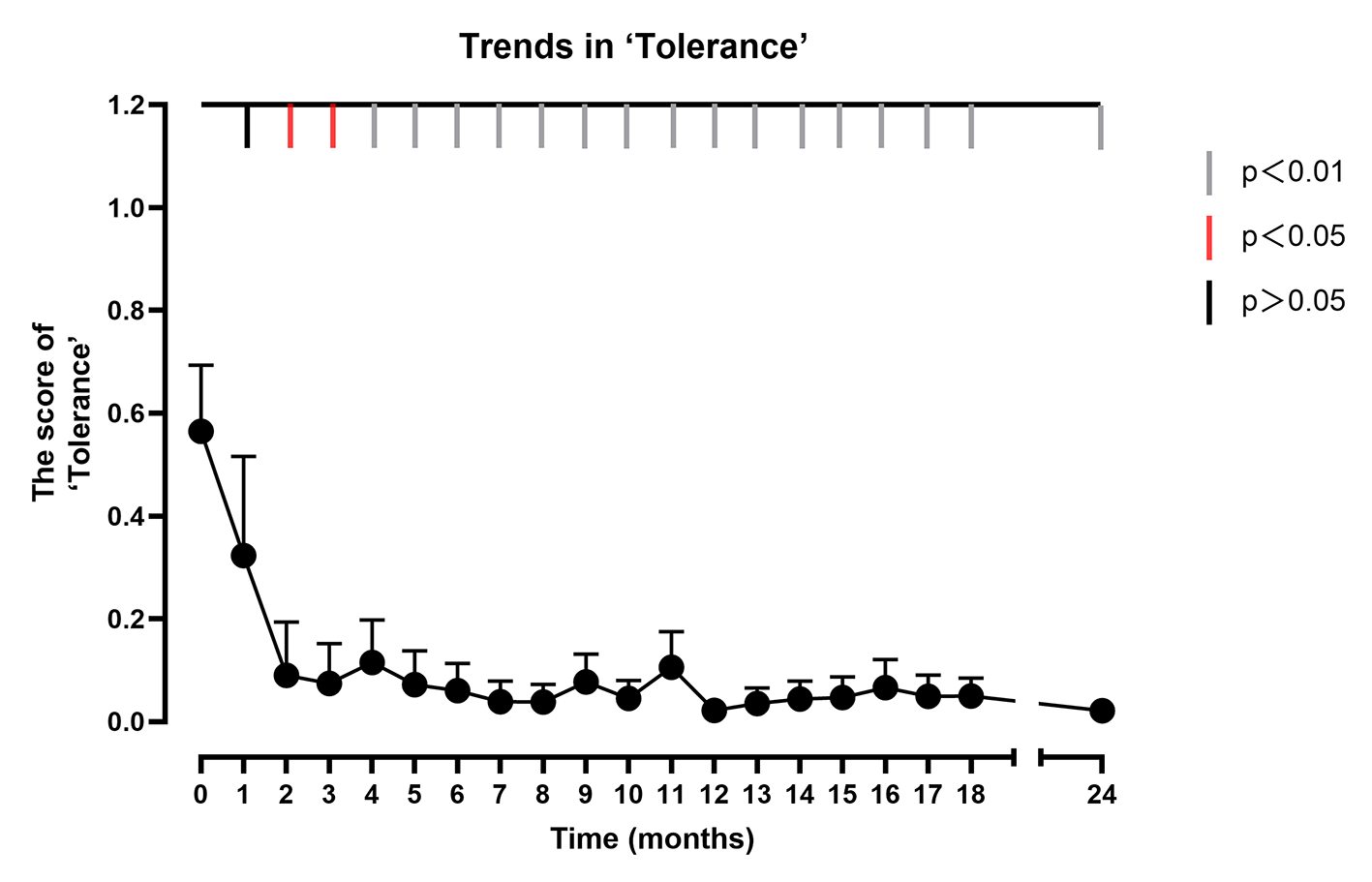

Supplement: SUPPLEMENTARY FIGURES S1-S11 — Trends in each symptom of the YFAS 2.0. [file Data_Sheet_1.zip › Image 6.TIF]

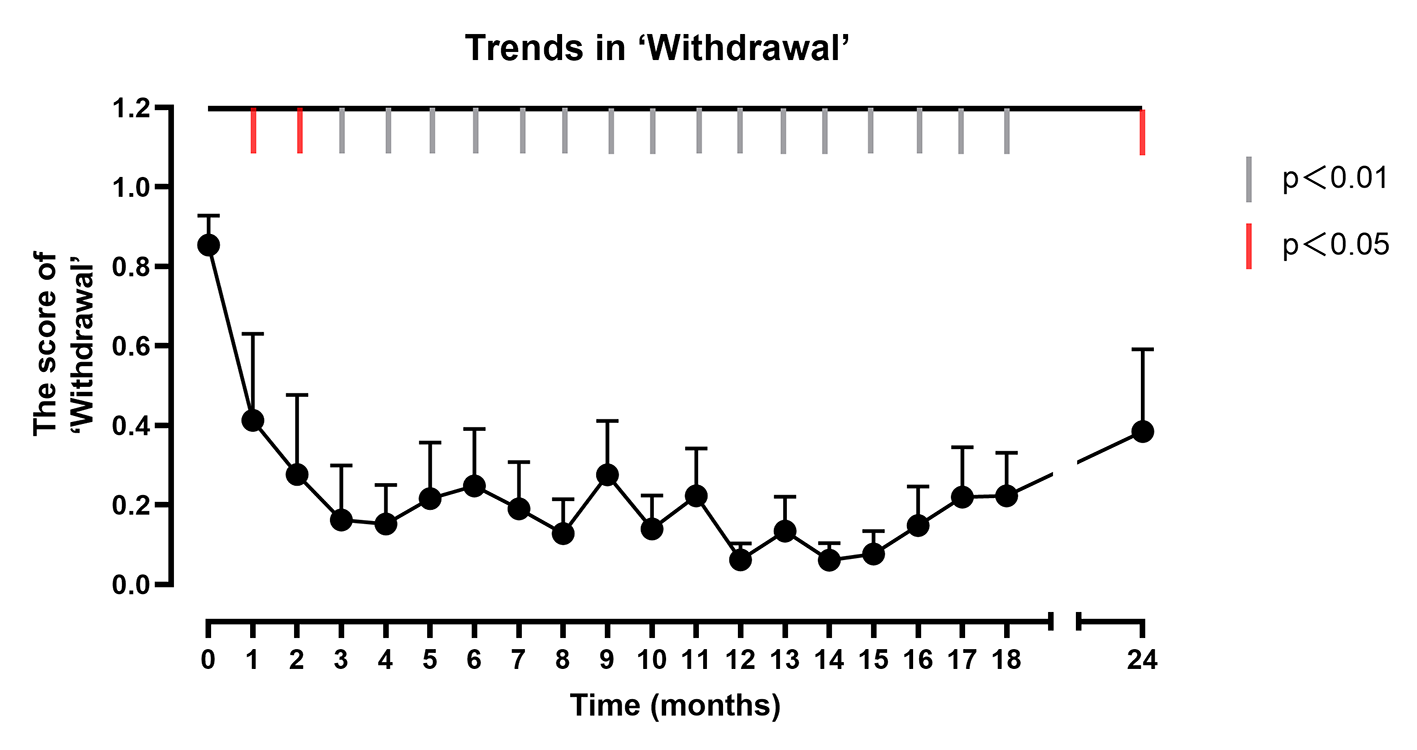

Supplement: SUPPLEMENTARY FIGURES S1-S11 — Trends in each symptom of the YFAS 2.0. [file Data_Sheet_1.zip › Image 7.TIF]

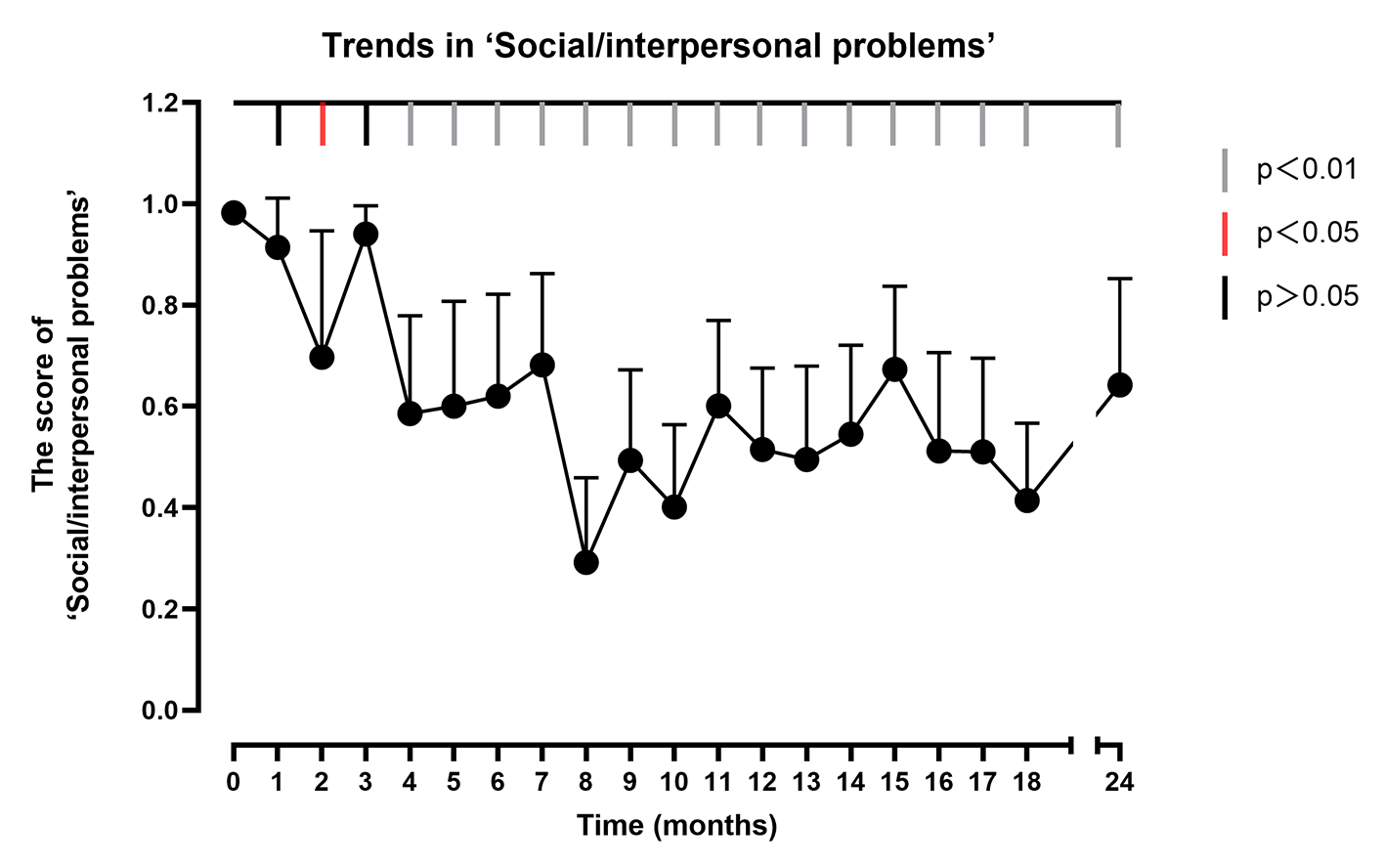

Supplement: SUPPLEMENTARY FIGURES S1-S11 — Trends in each symptom of the YFAS 2.0. [file Data_Sheet_1.zip › Image 8.TIF]

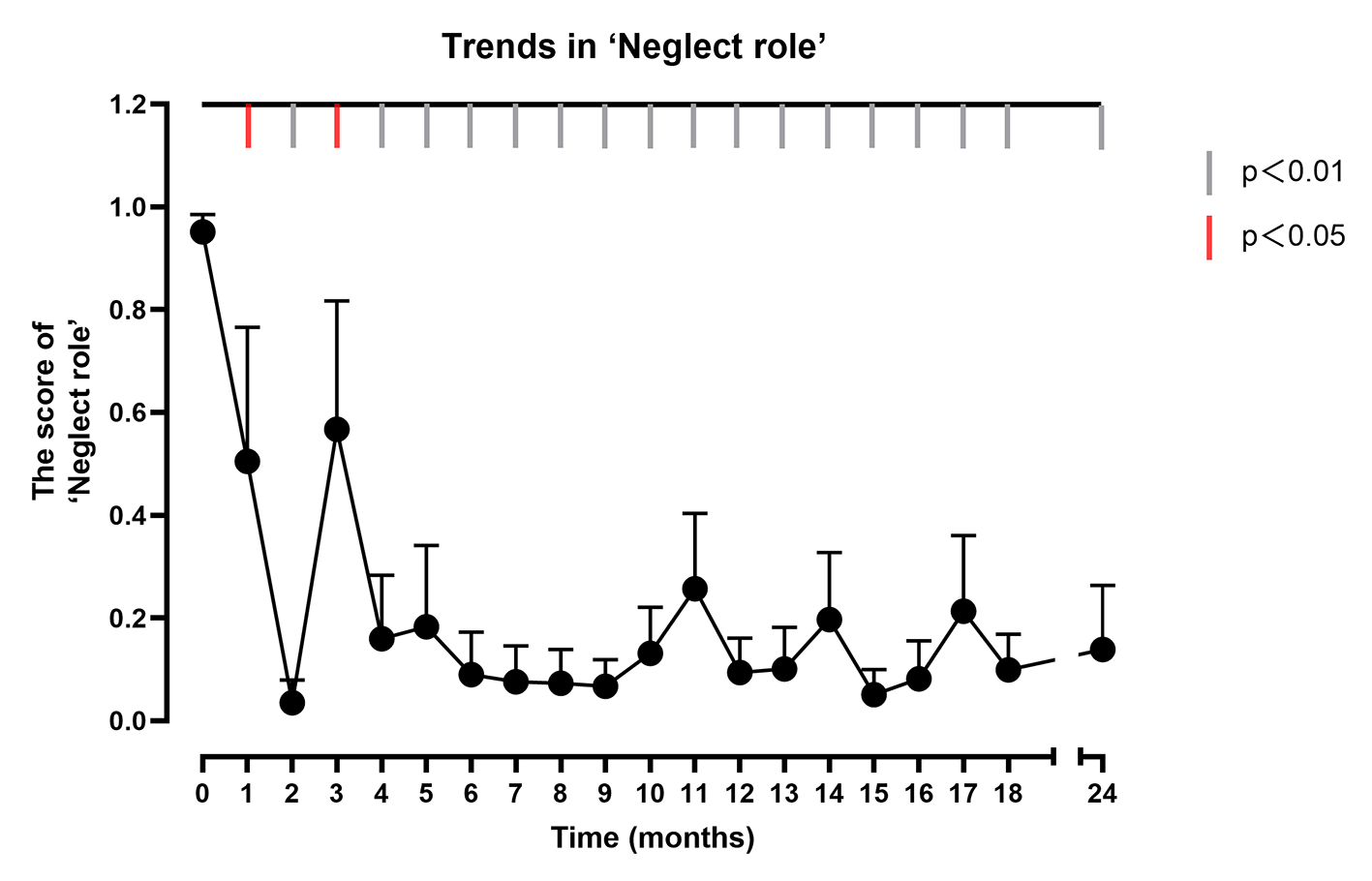

Supplement: SUPPLEMENTARY FIGURES S1-S11 — Trends in each symptom of the YFAS 2.0. [file Data_Sheet_1.zip › Image 9.TIF]
